# Exploring Trans People’s Narratives of Transition: Negotiation of Gendered Bodies in Physical Activity and Sport

**DOI:** 10.3390/ijerph18189854

**Published:** 2021-09-18

**Authors:** Sofía Pereira-García, José Devís-Devís, Elena López-Cañada, Jorge Fuentes-Miguel, Andrew C. Sparkes, Víctor Pérez-Samaniego

**Affiliations:** 1 AFES Research Group, Department of Social Work and Social Services, Universitat de Valencia, Avda Tarongers 6, 46022 Valencia, Spain; sofia.pereira@uv.es; 2 AFES Research Group, Department of Physical Education and Sport, Universitat de Valencia, C/Gascó Oliag, 3, 46010 Valencia, Spain; victor.m.perez@uv.es; 3 AFES Research Group, Department of Body Expression Didactics, Universitat de Valencia, Avda Tarongers 4, 46022 Valencia, Spain; elena.lopez-canada@uv.es (E.L.-C.); jorge.fuentes@uv.es (J.F.-M.); 4 Carnegie School of Sport, Cavendish 101, Headingley Campus, Leeds Beckett University, Leeds LS6 3QU, UK; A.C.Sparkes@leedsbeckett.ac.uk

**Keywords:** transgender, storyline, embodiment, exercise, active lifestyle

## Abstract

This paper explores how trans people who make transitions negotiate their gendered bodies in different moments of this process, and how their narrative storylines are emplotted in physical activity and (non)organized sports (PAS) participation. A qualitative semi-structured interview-based study was developed to analyze the stories of eight trans people (three trans women, two trans men, and three nonbinary persons) who participated in PAS before and during their gender disclosure. A thematic analysis was conducted to identify the patterns in the transition process and the structural analysis of the stories from the interviews. Three transition moments (the closet, opening up, and reassuring) were identified from the thematic analysis. Most participants showed difficulties in achieving their PAS participation during the two earlier moments. The predominance of failure storylines was found particularly in men, while success was more likely to appear in women because their bodies and choices fitted better with their PAS gender ideals. The nonbinary trans persons present alternative storylines in which corporeality has less influence on their PAS experiences. The knowledge provided on the moments and the stories of transition help to explain trans people’s (non)involvement in PAS and to guide policymaking and professional action in PAS fields.

## 1. Introduction

Social and medical transition are crucial for the development, consolidation, and affirmation of the gender identity of many trans people [1]. Transition is considered, according to Klein et al. [2] ‘the process during which a transgender person alters their gender expression to be consistent with their gender identity’ (p. 556). This includes many different aims and practices that contribute to their physical, social and mental health. Some imply little changes in the body, as changing dressing style, hair restyling, and/or changing names and pronouns preferences. Although this is not compulsory, many trans people adopt small changes and some choose to adopt medical procedures (such as hormones and surgery) to drastically and irreversibly change their bodies. As Smith et al. [3] have indicated, whilst well over three quarters of trans people pursue transition socially, around 40% do not wish to pursue medical interventions, particularly those who are nonbinary. Along with these changes, many trans people embrace masculinity or femininity, as trans men and trans women do, while others do not identify themselves as trans persons and prefer the term ‘nonbinary’ instead of binary terms; they prefer to identify themselves as agender, genderqueer, gender fluid, or nonbinary persons. In fact, cohort research repeatedly shows a significant increase in the proportion of the youth who identify themselves as transgender or gender diverse persons in the last decade [4]. Around half of these young people identify as nonbinary [3]. Echoing this diversity, in this paper, we comprehensively use trans person or trans people as an umbrella term to name individuals or groups whose gender identities do not match their assigned sex at birth [5]. The diversity comprised in this term foreshadows that, far from being a homogenous or straightforward process, several understandings of transitioning can coexist, providing an awareness of the complex social interactive process encompassed in the negotiations of gender [6].

Although not all transition processes take place in the body, bodies play an important role in the negotiation of gender for those who decide to transition. In this paper, we consider how transition processes are inscribed in the bodies of trans people who participate in physical activities and (non)organized sports (PAS) practiced in different environments such as gyms, sports fields, locker rooms, beaches, pools, or dance studios. The processes of gender negotiation enacted in these environments are dialectical in nature, as trans people and social environments inherently learn from each other [7,8]. As Budge et al. [9] state, trans persons see themselves mirrored in the eyes of others as those eyes see themselves. While the emotional, psychological, physical, and sociocultural components of gender negotiation in transition processes are unique to each person and need to be explored on an individual basis [10], it should be taken into account that gender negotiations take place within structural constraints that influence patterns of behavior.

PAS contexts are characterized to a great extent by cisnormativity and sex segregation structures that traditionally hinder the participation of trans people [11]. Several studies refer to the wide variety of barriers and transphobic discriminations that trans people experience in PAS [11,12,13]. López-Cañada et al. [14] found that gender disclosure negatively influences trans persons’ engagement in social PAS, probably to avoid discriminatory situations and rejection in these environments.

Against this backdrop, the purpose of this paper is to explore how trans people negotiate their gendered bodies in different moments of their transition processes. A consistent understanding and appropriate knowledge regarding their experiences in these contexts seems paramount for professionals and politicians to identify transition care needs and policies to support trans people in PAS and promote a sort of PAS that could enrich their lives [2].

## 2. Narratives of Gender Transitioning Bodies and Sport

To fulfill this aim, we adopt the lenses of narrative inquiry. According to Sparkes and Smith [15], people give meaning to their lives by telling their ‘stuffness’ to others and themselves. As bio-social narratives, stories include ‘bodily sensations and experiences as it is the body that carries the experiences that are the topic of the stories. Within this process, the corporeal character of the body as an obdurate fact shapes the stories that come out of it’ (p. 359). Although people live unique corporeal and biographical experiences, the kind of stories they tell are inevitably influenced by the kind of body they have. These bodies and their narratives, in turn, are shaped by socially shared conventions of reportage and story hearing and reception [16].

Given the views expressed by Sparkes and Smith [15], it is interesting to note that to gain a better understanding of trans persons’ experiences some scholars have focused on their embodied transition narratives. For example, in Prosser’s [17] book, *Second Skins: The Body Narratives of Transsexuality*, the process of narration and the available stories of trans people in cultural settings are seen as an essential resource for those who want to modify and re-gender their bodies. In this case, the desire to achieve a different final gendered body is required to gain social acceptance and medical authorization to transition. These narratives reinforce the model of the ‘wrong body’, focus its discourse on the genitals, and determine that gender disclosure necessarily means a quest for feminizing or masculinizing bodies to the full extent [18]. As temporality is interwoven in narratives, these changes require direct transformations without ‘looking back’ to reveal the real gender as soon as possible [19].

In narrative terms, the transition can be considered as a process of emplotment gender identities. To emplot, Frank [20] argues, is to connect things in a relationship of mutual, sequential effect, determining that one thing happens in consequence of another. Narratives provide sense and direction to personal story plots. Even though the wrong-body narrative of transition has been dominant in western societies, nowadays it is being questioned for several reasons. First, it does not consider the richness of transition processes and the multiple meanings that trans people may give to their gender identities over time [21]. As Israeli-Nevo [19] points out, not all trans people opt to embrace an exclusive transformation from one gender to another, thence automatically breaking with their past identities. Some prefer taking their time while transitioning to explore alternatives and, eventually, taking on board some of their past identities to smoothen a timeline coherence. Second, many trans people that defend the fluidity and unfixity of genders are not concerned about fitting into male or female social roles, and struggle with female/male binarism and continuum [22,23]. In summary, these arguments sustain the idea that transition processes do not mean that trans people have to correct their ‘wrong bodies’ but instead gain awareness and learn how to re-emplot their embodied gender identity.

This new understanding of transition processes, however, has hardly been addressed in PAS domains. Travers and Deri [24] and Sykes [13] examine how trans people’s storylines are emplotted in PAS participation to narratively develop their gender identities as desired. Organized physical activities (in particular, team sports) play an especially relevant supportive role for those who are living through a disclosing process [25,26]. Their experiences, however, differ substantially from one person to another as they are involved in very different dynamic and complex outness processes [27]. Elling-Machartzki [6] studied a group of 12 transgender persons in three stages of the transition process (pre-transition, liminal and post-transition phase), showing the rigidity of gender categorization in these contexts and the lack of a policy addressing the recognition of body multiplicity, especially in bodies that are involved in transition processes. This single study represents a key effort in analyzing PAS from a non-essentialist gender perspective and locating trans people’s experiences at the core of the discussions which question the current gender order. Therefore, it is timely and convenient to delve into the challenges and gender negotiation processes in PAS settings within other socio-cultural contexts, as well as complementing conventional ways of content analysis with other forms of narrative lenses.

## 3. Materials and Methods

### 3.1. Participants and Procedure

This study is part of a larger quantitative–qualitative research project on trans people’s PAS participation, their experiences, and other psychosocial issues. In the first quantitative phase of this project, a total sample of 212 Spanish trans people completed a survey on their PAS participation (see [28]). Access to potential participants was obtained through LGBT associations and gender units from different hospitals. A snowball strategy was then adopted to widen the number of participants voluntarily engaged in the research. The participants were offered the opportunity to be interviewed in a second qualitative phase to obtain further insights into aspects related to their PAS experiences. From those who voluntarily expressed their willingness to collaborate, and for the specific goals of this research, eight participants were selected according to the following criteria: (a) All the informants participated widely in PAS before and after they disclosed their gender identities, in different contexts (recreational, professional, and/or educational link); (b) participants self-defined as nonbinary, who were rather a minority group in the quantitative sample, were prioritized in this qualitative phase to widen the range of different narratives of transition. (c) Based on the contact with the sample in the first quantitative phase, participants identified with potentially rich stories were privileged. Details of the participants are provided in Table 1.

Qualitative data were gathered through individual semi-structured interviews that lasted between 45 min and 1 h. The aims of the research project and the purpose of the interview were explained in a previous telephone call, and an agreement was made to meet at the interviewees’ convenience. The participants had the opportunity to express any doubts and ask any question they considered necessary. The interviews were audio-recorded and fully transcribed verbatim. Some examples of the interview questions were:

*“When did you first publicly express your gender? How was your disclosure socially received? Are you currently comfortable/displeased with your body? Which parts of your body do you like the most and the least? Have you faced obstacles as an athlete or just the opposite? How did you deal with them? Did your teammates know your trans identity? When did you tell them? Why?”*

### 3.2. Ethical and Validity Concerns

The research project and the materials and procedures used in this paper were approved by the Ethics Committee of the Universitat de València. Participants signed an informed consent form to authorize the research team to publish the data. Beyond these formal conditions and the use of pseudonyms for anonymity, as a research team we dealt with ethical dilemmas that emerged during the ongoing fieldwork process. We thus adopted relational ethics, which emphasized mutual respect and connectedness between researcher and subjects [29]. Following Braidotti [30], we were concerned about telling the difference between a non-unitary voice or a single story. This is because, as shown in Table 1, the participants self-identified differently in more than two binary categories.

We recognized that our own bodies and gender identities were important to those of our participants and constituted a key dynamic factor that permeated the research process [31]. Accordingly, we negotiated our cisgender status (non-trans) and the way we presented ourselves to the participants as we managed our femininity or masculinity in front of them whilst avoiding exaggerated gender expressions of either. These actions were not intended to be associated with heteronormativity, which could negatively influence the process of data collection. During the interviews, our actions were also limited by the sensitive position of our participants regarding the transition process and their feelings about their bodies. We prioritized respect and care for the participants over our research interests, which meant omitting questions that participants might find intrusive or upsetting. For example, we avoided asking how they felt with their bodies when we found they were struggling with strong gender dissonance and perceived them to be emotionally affected by this during the interviews.

Besides ethical concerns, in terms of validity, the study was underpinned by the criteria presented by Tracy [32] to provide quality to the research process and findings. One criterion was member checking since the transcribed interviews were returned to the participants for reading, modifying, and extending their views if they considered it necessary, as well as obtaining their explicit agreement to use their data. We also used a triangulation of perspectives from different trans people (women, men, and nonbinary persons) and their transitions to achieve multiple views and voices on the complex issue of their gender negotiations during the transition process. We did our best to be transparent and sincere during the writing of this paper, as well as being respectful to the LGBT association and collectives that allowed us access to the participants by sending them all our research results and collaborating with them in its public dissemination.

### 3.3. Data Analysis

Our qualitative data were first subjected to thematic narrative analysis. According to Riessman [33], the main focus of this analysis is the content of what is said in stories, rather than ‘how’, ‘to whom’, or ‘for what purposes’. The first author was involved in a process of familiarization in which she deeply immersed herself in the data by undertaking multiple readings of the interview transcripts to become intimately familiar with their content. Following this, as recommended by Braun et al. [34], she engaged in the coding process that involved carefully reading the data and ‘tagging’ each piece with a code that had some relevance to the research question of how the participants experienced transitioning in PAS settings. Once this coding was completed, she organized the codes into basic and organizing themes, discussing with the research team the emerging patterns of meaning concerning transition processes, as shown in Table 2.

Following the thematic analysis, a structural analysis was also conducted on the interview data. As Riessman [33] noted, the structural analysis shifts attention from the told to the telling, focusing on how the story was told and organized or scaffolded within certain plot lines or scripts. Thus, the questions asked were: What kind or types of stories do the participants tell about their experiences of transitioning in PAS and how do the types of stories they tell shape their experiences? Here, our analysis was informed by the work of Gergen [35], who considered that every plot or structure developed in time could adopt a linear form, considering evaluative changes in the story. The narrative sequence and the events selected by the storytellers showed how stories had been put together and clarified their final points. In this process, the events described by the narrators moved them closer to, or away from, their goals.

Gergen [35] proposed organizing relevant events into Cartesian coordinates’ axes in which linear stories in time were drawn in a two-dimensional and evaluative space. Although multiple narrative lines were possible, he suggested three basic forms of storylines: positive, negative, and stability lines. When the story moves toward a successful end, victory, or award, the storyline over time became positive. On the other hand, the storyline became negative when it was directed toward a negative ending, a failure, or a sense of disillusionment, while stability narratives were produced in stories in which storytellers’ trajectories remained constant, without remarkable evaluative inflections.

Given the kinds of analysis undertaken as described above, we presented our findings in two sections. The first section reflected phases of the transitioning process over time. The second section reflected the evaluative dimension of different narrative structures.

## 4. Findings and Discussion

### 4.1. Transition Process in Time

Transition processes are inscribed in time. However, as we have noted above, the idea that gender transition can be clustered together under a linear time process is problematic on many fronts. Not all trans people have the same conception of femininity or masculinity, and not all trans people agree on the body changes needed to fulfill the expectations of their genders. As an iterative and non-linear process, gender transition ‘has no prescribed beginning and end’ (p. 3811), according to Haimson et al. [36]. That said, it is obvious that time counts in storytelling about transition processes. Israeli-Nevo [19] points out that trans people’ stories of transition are paced by turning points characterized as temporal thresholds that mark a divide between ‘before’ and ‘after’, as well as the ‘past’ and the ‘future’. Against this backdrop, our depiction of the transition processes is organized by time into three stages labeled ‘in the closet’, ‘opening up’, and ‘reassuring’. These stages are dynamic and can be traversed with pauses, advances and retreats influenced by social and individual questioning and expectations [1]. Therefore, despite these stages being presented consecutively, people who choose to transition may not follow linear transition paths. It is important to remark that this narrative structure is not related to any prescriptive or predetermined desirable outcome. It is simply aims to encapsulate the particular events, attitudes, and practices contained in the participants’ storytelling in their transition over time.

#### 4.1.1. In the Closet

Many trans people’s experiences are characterized by keeping their gender identity as a secret from their family, friends or other persons around them [37]. According to Brumbaugh-Johnson and Hull [38], the way they navigate general cultural expectations, the threat of violence and the anticipation of others’ reactions influence their decision of who to come out to and when to do so. Being in the closet is a strategic decision which anticipates the potential risk and level of safety which can occur long-term. Nowadays, coming out, for multiple reasons, may not be a viable option for many people and others only decide to come out and transition when their feelings of gender dissonance are too strong [1]. However, as Rasmussen [39] and Sedgwick [37] suggest, being in the closet does not mean being disempowered and people who come out are not necessarily more empowered. The risks of considering this simplistic view of the closet and coming out discourses should not be privileged or imperative for trans persons [37,39].

Remaining closeted or coming out is represented normally dualistically: in/out, public/private, before/after. However, the closet is not a time-limited period and cannot be fully accomplished since new closets are erected in each new context and encounter [36]. Trans persons may manage these situations throughout the transition period, but most experiences in the closet occur before transitioning, when they are still exploring gender. For many young people, this is especially an anxious period involving a complex process of self-knowledge and self-acceptance in which they feel uncomfortable regarding their assigned sex [1].

The participants’ stories in PAS contexts reflected their personal and social conflicts, especially those experiences in which they felt compelled to behave according to their sex assigned at birth rather than their gender identities. Many participants who self-identified as men or women preferred to practice ‘male’ or ‘female’ PAS that culturally matched with their gender identities. Male participants usually refused to play team sports considered too ‘girlish’, though sometimes they were forced to take part in these sports. For example, Carlos felt impelled to play volleyball with girls, a sport he loved, instead of football with boys. Female participants instead mostly desired ‘girly’ exercises, such as ballet, which was Carolina’s preferred activity, and rejected ‘male’ activities, as Ana recalled: ‘I didn’t hang out with the boys because they were beasts and only played football (…) and I don’t like football’. In contrast, nonbinary participants were more flexible toward female and male PAS manifestations. For instance, PE allowed Daniel to release all his energy through competitive sports and games and engage in dance, which he greatly enjoyed.

While in the closet, some participants were perceived by their peers as outsiders. At school, classmates used to taunt and tease Daniel, Iker, Alex, and Raúl with insults like ‘tomboy’ and ‘hairy Mary’. As Daniel explained, ‘the more masculine I was, the more insults I got’. For Carlos, his very desire of playing ‘inappropriate’ PAS was also a cause for rejection: ‘the teachers saw that they were hitting me at recess (…). They did absolutely nothing and washed their hands, I was apparently the culprit of everything, because I was the one who wanted to play football’. Moreover, Ana, Carolina, and Llurena felt marginalized, rejected, and bullied at school for being the ‘queer’ ones, especially in PE: ‘Alone, isolated, marginalized and bored to death [in PE]. The most graphic example was to be the last to be chosen in gym groups’ (Ana).

During childhood and adolescence, participants frequently had to negotiate their access and participation in PAS with adults in disadvantaged power relationships. For instance, Alex, Raúl, Carlos, and Llurena felt oppressed when their parents forced them to participate in undesired PAS activities. Alex and Carlos’s mothers were constantly reinforcing their female role, something that irritated them the most:

*“My parents have never come to see me in all my exams, including judo and taekwondo and my competitions. Yet, when they forced me to do all that gymnastics shit, wearing that bloody leotard… my mum used to come saying ‘I am so proud’, as if they were supporting me, which I hated. I felt humiliated by their support.”*

(Alex)

*“My mother’s desire was for me not to do anything that was ‘tomboy like’ as she said. My mother knew that I let off steam in physical education and she wanted to cut that.”*

(Carlos)

Many participants also felt anxiety and insecurity in very cisnormative PAS spaces, such as locker rooms, beaches, and swimming pools, in which they inevitably had to uncover certain body parts to others [6]. Before coming out, these spaces were seen as potentially dangerous and often avoided, as Carolina expressed about beaches:

*“I have denied or tried as much as possible not to go to the beach, for example, because of the swimsuit. (…) I have to wear this swimsuit, and I have to go with a bare torso and I do not have breasts. It is a little shameful to be a woman and not have breasts.”*

In these places, the participants were more conscious and sensitive about their bodies and many presented narratives of body–self disassociation in which they rejected their own bodies and were self-excluded to avoid anxiety and threats to themselves [6,13,40,41]. This sense of body–self dissociation did not only increase in public spaces. There were situations in which these participants had to struggle with undesired body characteristics that strongly negated their gender identities, such as menstruation (and therefore, a ‘female’ body) in the case of Raúl: ‘I stayed in bed, crying. It hurts your soul [when you menstruate], because it defines you and reminds you every month you are a woman’. In this case, the body–self dissociation mediated by menstruation also affected PAS since it discouraged him from playing football during this stage.

#### 4.1.2. Opening Up

Gender disclosure can be gradual or abrupt, it can happen on a particular day, or it may be extended in time. Anyhow, many trans people situate their public coming out as the turning point that begins their transition. Typically, thereafter, changes in appearance become progressively more noticeable. In the beginning, bodies are more androgynous. At this early moment of their transition, most participants recognized that avoiding being detected as a trans person became their foremost concern. The main reason was to prevent harassment and other negative situations that usually occurred in PAS cisnormative environments [14,42]. Therefore, participants adopted hyper surveillance attitudes over their bodies and ’passing’ strategies to present gender normative bodies to others [43,44,45]. This could be seen in Llurena, who controlled the visibility of certain parts of the body, namely her Adam’s apple, to minimize the depiction of masculine features beginning stage. Ana also stuffed her bra and used a girdle to feminize her figure. These accessories were uncomfortable to wear and hindered her ability to run in PE activities. She actually did not enjoy PE as she ‘had to choose sporting outfits that made me[her] masculine’ or wearing tights that marked her ‘eyesore’, as she called her penis. Daniel and Alex also feared their breasts would be noticed in the gym. Accordingly, even though the tight-binding they used to flatten their breasts in PAS impeded their breathing, they chose to do so because they felt that the uncontrollable and undesired movement of their breasts would threaten their newly acquired masculine identities. As Alex stated, ‘I have to do it [exercise] with a sports bra and my boobs come and go, and then it is an uncomfortable issue, so the more invisible and hidden they are the better’.

These stories reflect how body changes and body strategies were adopted by many participants according to a ‘self-as-normal’ narrative, where being ‘normal’ means to look and behave like a ‘normal’ man or woman. According to Planella and Pié [46], in achieving such normalization the assumption is made that feminine or masculine bodies are required to practice PAS as women or men. At this early moment of transition, however, this narrative conflicts with the actual ‘freakish’ androgynous appearance of the bodies involved. As a result, most of the narratives of these women and men depicted them as abjected, without the possibility of finding a way of being themselves in ‘straight’ gendered spaces. Sometimes, participants felt that the strategies of ‘passing’ adopted were insufficient or inappropriate to safely occupy these spaces. For instance, Raúl kept away from beaches because his gendered body reflected an illegible unnaturalness: ‘Imagine my body with hair (…) how am I going to be covered from above and with a male body? (…) it is not conceivable’. In this location, PAS participation reinforces a finalist sense of transition, whereby steps forward become apprehended as advances toward right bodies, that is, normative binary bodies.

However, some participants eventually developed alternative narratives in non-cisnormative PAS, namely the participation in LGBT teams or spaces that provided support and a sense of belonging to non-normative transitions [6,47]. For instance, LGBT-friendly beaches offered Daniel the opportunity to live openly as a non-cisgender person. Llurena participated in an LGBT basketball team because she wanted to practice with other different women, and Iker chose to swim in a sports center due to the sort of people he could find there:

*“Some girls are six inches taller than me, with my same shoe size (...) and it is reassuring or to find a lesbian who is also a tomboy lesbian...there is scope for action”*.

(Llurena)

*“I like to go to a place where I see fat people, hairy people, and where I can see Pakistani and Chinese persons in the jacuzzi... and me there. I like that, it makes me feel good”*.

(Iker)

Other times, participants created alternative narratives by questioning gender cultural practices they refused to assimilate. This was the case of Iker, a transgender man, who found some male behavior senseless. For instance, he did not recognize himself in certain behaviors he witnessed in male changing rooms:

*“I went to the [male] changing room and I felt uncomfortable (…). They did not talk to each other, but they walked around showing off their dicks to each other and it made me feel a little uncomfortable.”*

#### 4.1.3. Reassuring

At some moment, the conceptions of gendered body–self disassociation that have been negotiated along the transition processes start reassuring. Many participants reaffirmed their gender according to the embodied sense of self-acceptance and social recognition of their identities. This is a process of gender performance that happens in different spaces. Although gender transition does not necessarily entail an end in terms of achieving a particular body, in their accounts most participants told body adequacy stories of succeeding in being identified as cisgender women or men. In these cases, they felt the benefits and the subjective sense of security that came with completely passing, that is, to go unnoticed as a man or woman, instead of a trans person. Llurena, Carolina, and Carlos told adequacy stories after adopting multiple medical procedures to change and re-gender their bodies, including hormone treatment and surgery. Carlos underwent several mastectomy operations, while Llurena had a vaginoplasty and Carolina had breast enlargement surgery.

In PAS contexts, the straightening of bodies may enable the trans participants to have increasing experiences of safety and emotional ease [6,45,48]. In this regard, Carlos, Carolina, and Llurena’s surgical body modifications contributed to their acceptance and inclusion in PAS participation. Carlos recognized that after a mastectomy he gained self-confidence in gyms by not having to hide his (female) breasts: ‘Now I’m more social, I feel better, I notice it, I feel better with myself as long as I do not have to hide myself anymore’. Carolina’s ‘new body’ and ‘the new use’ of it after gender-affirming surgery provided her with the courage and confidence to occupy gendered spaces that were previously not accessible to her, such as locker rooms: ‘At first I covered myself scantily, that was my passport, my body was my passport to be legitimized anyway if someone had any doubt’. Likewise, Llurena also felt good about sharing locker rooms with cisgender women after gender-affirming surgery:

*“It was incredible, [before surgery] I had always showered in three minutes. Additionally, this time a mate girl came and asked if I was going to go out of the shower because I’ve been inside for fifteen minutes. I thought it cannot be true, fifteen minutes! (…) I walked out of the shower very calmly, it was a stillness I wasn’t used to.”*

Scattered between these reassuring experiences is the strong gender regulation exerted in PAS contexts that blur end-up stories. For instance, Raúl was forced to withdraw from his female sports team for testosterone consumption, while he could not compete in a male team either, due to the legal prerequisite of changing his National Identity Card sex category to participate in this team as a man. As Lucas-Carr and Krane [49] maintain, ‘there is no space for athletes who are transitioning to continue participating in competitive sport’ (p. 539). Raúl also feared that as time went by his chance of standing out became more difficult: ‘With the age that I am, I won’t reach a high standard [playing with the boys]’. For him, complete passing was hindered by the impossibility of reaching high-performance in a male football team or not being ‘good enough to compete’, like Bryan, another elite trans man athlete also reports in Klein et al.’s study [2] (p. 563).

Nonbinary participants’ bodies brought into focus different reassuring stories. They regarded body changes as optional, not imperative, to achieve self-acceptance and inclusion. Thence, at this stage, their stories of transition were not bound to passing or medical interventions. Instead, they focused on body acceptance and getting along with their bodies:

*“[The transition process] has been a personal achievement and I did it by myself without [medical] help.”*

(Daniel)

*“I feel good with my body (…) my body will always give me the maximum sensitivity and is not to be operated (…) and I do not bind [my chest].”*

(Alex)

In fact, although they opted for male names, neither Daniel nor Alex struggled to achieve masculine body features in any way. Additionally, both participants felt their gender identities were reassured by their nonbinary appearance.

### 4.2. Storylines

Transition stories are not only stories about bodies. Actually, as Frank [50] points out, they are stories told by and through bodies. In fact, along with their ongoing process of becoming, trans people’s bodies provide a sense of direction and wholeness to transition storytelling. The structural analysis of the participants’ stories generates, according to Gergen [35], storylines of success and decline which provide consistency to their experiences over the transition. The actual fleshy reality of the body in transition is the axis around which the evaluative dimension of the storytelling on transition gravitates.

In this study, the stories of success entail full inclusion and acceptance in PAS environments. In the closet and opening up stages, participants’ stories center on the many obstacles and barriers that their bodies encounter in PAS at that moment, as well as their struggles to overcome them. For most participants, positiveness is related to the extent their bodies fit with socially suitable gender identities. This is the case for the trans women who succeed in passing and consider that occupying female PAS and facilities as women was a personal triumph. Developing a curved and non-masculine body and achieving social acceptance in female sporting contexts and practices are the essential PAS contributions to their stories of a successful transition. PAS helped participants to succeed in their transition insofar as they fostered body changes and social recognition according to womanized ideals. Carolina exemplified this narrative of success when she spoke of her achievement in using changing rooms and working out the gluteus and leg muscles to tone her body up ‘as the rest of the women do’.

In contrast, the stories of failure tell participants’ PAS experiences of exclusion by the inability of bodies to do what they should do according to gender binary ideals. This storyline is especially seen in trans men narratives. Even if their appearance is rather ‘masculine’ when they do not achieve the sports performance required for a male body in competition, they fail to be fully included in PAS. This was the case of Raúl and Carlos, whose mediocre performance in male teams contrasted with their previous professional level in female football teams before their transition. Thence, their stories of failure are emplotted in bodies that are insufficient to play in male teams. The obstacles they found hindering them from achieving a full integration into male sports made it difficult to construct and perform a masculine social identity and impeded their successful transition in sport contexts. In contrast, when inscribed in non-normative bodies, success and failure were not dependent on specific body transformations and did not entail passing. Instead of seizing the possibilities and struggles posed by bigenderism in PAS contexts, their lines of failure or success were less sharply defined by the accommodation of embodied gender ideals.

Thus, in their transition, trans people are called on to assume or reject particular forms of femininity, masculinity, or queerness drawing from stories available in their socialization contexts. Over the years, they develop narrative habitus that affirm and represent who they are and who they ought to be. The concept of narrative habitus is useful for understanding the collective dimension of transition stories that underlie these experiences of failure and success shown in our study. According to Frank [20], the narrative habitus involves the embedding of stories in bodies in ways that prompt people to focus on ‘some stories like those that one ought to listen to, ought to repeat on appropriate occasions, and ought to be guided by’ (p. 53). The way people feel in response to stories depends on the embodied sense of attraction, indifference, or repulsion fueled by their narrative habitus as an undriven force in choosing a story that represents a world they feel they belong to. In this sense, our participants develop, often tacitly, a perception that a certain story is (or is not) for them.

Almost unavoidably, trans people’s embodied stories demand a certain anticipation of how they should end [2,50]. These persons envisage themselves in the future delineating plotlines with an ending that is directly connected to their transition trajectories. What Frank [20] denominates as predictable plot completions precisely allude to people’s ‘sense of a correct and fitting resolution toward which a half-told story should progress’ (p. 54). In transition stories, plot completions are enacted according to embodied expectations and possibilities of transformation. These social expectations and aims on their gendered bodies serve as the fulcrum that positively or negatively slopes the storylines of many participants. For instance, some complete the plot line projecting onto the future the stories they hear and imagine success stories in the advanced stages of transition which entail radical interventions to mold their bodies to gender binary expectations. In those cases, participants, who have not undergone mastectomy or gender affirmation surgery, assume the benefits that re-gender surgeries entail, therefore expecting to undergo these procedures in the future. This is the case, for instance, of Daniel and Raúl, who believe that a mastectomy would allow them full and unrestricted access to PAS environments, including locker rooms. Moreover, these examples show how going through masculinization processes, those connected with PAS, led most trans men to improved mental health since a majority of trans men attributed their suicidal ideation, self-harm and attempted suicide to personal issues related with their masculine gender identity [51].

In general, the storylines created by the participants in this study are strongly influenced by the binary sex/gender system. This system lies at the heart of the self-as-normal narrative, acting as an essential narrative resource to provide a sense of coherence and identity continuity to transition stories. Thus, the participants’ abilities to fit their bodies to the self-as-normal narrative becomes paramount in the storytelling of their experiences in PAS, drawing different stories of success and failure. Success is more likely to appear in the stories of women whose bodies and choices better fit with their feminine ideals, while failure is predominant among the stories of men who desire to compete in sports.

However, not all trans people adopt a self-as-normal narrative and a stable plot line because, according to Frank [52], they are ‘rarely docile selves who passively accept any story’ (p. 430). Storytelling entails the agency to search for meaning and identity it in relation to other storytellers [53]. Therefore, trans persons do not necessarily stick to one narrative, but swap different narratives in creative ways, adjusting them to their circumstances in the process of constructing a consistent narrative of the self that sustains and motivates their transition processes.

In this study, this is particularly the case for nonbinary people’s stories whose storylines are very ambivalent and unpredictable. They depict both regressive and progressive directions, combining non-passing and passing accounts that sometimes conform to, but also challenge, gender dichotomies. For instance, Daniel’s story illustrates this point, as he retreated from gyms due to his androgynous body but decided to make this body visible in other public PAS spaces, such as beaches, which he transformed into a territory of subjective resistance [44]. Through these actions, Daniel positions himself in the present, challenging the idea of transition itself. Arguably, the conception of trans persons’ transitions as projections that can be told as stories is questionable, making obsolete the traditional linear conception of gender transition. Instead, a focus on the present symbolizes an ever-changing body always in transition.

## 5. Conclusions and Final Comments

This paper explores the PAS experiences of trans people in connection with their transition processes. The identification of separate transition stages in the closet, opening up and reassuring allows for the identification of key moments in the gender negotiation process. The results reveal how PAS participation influences and is influenced by the negotiation of the gendered body in transition in time. During the closet and opening up stages, trans people struggle with the gender roles associated with their sex assigned at birth. Later, in the reassuring stage, self-as-normal narratives drive trans persons’ efforts to occupy gendered PAS spaces and practices, as well as to be recognized by their gender identities, as in all the spheres of their lives. Most adopt several passing strategies to occupy cisnormative PAS spaces, while some others opt for non-normative sports teams.

Overall, the results reveal the complex and evolving processes of negotiation trans people have to deal with to participate in PAS. The binary sex/gender system greatly determines the narrative resources available for trans participants to tell their transition stories. The difficulties in transforming their bodies according to their gender ideals partly explain the predominance of failure storylines, particularly in male athletes whose new expressions of body–self cannot or are unable to fit into male sports domains. Failure also predominates in the storylines of nonbinary people, although success is also recognizable in stories which are less characterized by passing in non-cisnormative contexts. Success especially appears in those trans women whose bodies and choices better fit with feminine ideals.

However, we urge caution in interpreting these findings. Although the self-as-normal narratives are predominant in our participants’ stories, it does not mean that such storytelling accurately represents the whole trans community. Further studies including a greater diversity of participants, especially nonbinary persons, would be desirable to explore alternative narratives of transition. This study may also have overemphasized the importance of corporeality in trans persons. According to some authors, an exclusive focus on the body surface produces the denial of transition complexity [21,54,55]. Even avoiding a reduction in gender to what is visible, other possible factors, such as self-confidence or social support, should also be taken into account in the future. Finally, although useful analytical resources provide a sense of direction to storylines, the notions of ‘failure’ and ‘success’ may be too simplistic to comprehend the complexity of stories and real lives. In our characterization of failure and success, we must take into account that gender normative ideals act as a backdrop for most of the participants’ evaluations of their transition processes.

With these caveats in mind, we conclude that transition storylines are relevant to understanding trans people’s experiences in PAS. Accordingly, this paper offers relevant information for further interventions. In particular, it must be taken into account that failure storylines hinder their engagement in PAS, and impede participants to achieve their potential social, psychological, and biological health benefits. In most failure narratives, the inclusion of trans people in PAS relies on their obligation and responsibility to fit into the binary sex/gender system through a proper gendered image presentation and behavior. This can be unfair for those persons who refuse or cannot find a transition process to change their bodies to social gender ideals. Coaches, sports managers, health professionals, social workers and policymakers should assume their responsibility in promoting the development of sport, educational measures and policies to generate safe spaces in which all trans people can enjoy these activities and benefit from their participation in PAS, regardless of their transition stage or corporeality.

## Figures and Tables

**Table 1:** Overview of interviewees’ genders, age and information about their medical treatments.

Name (Pseudonym)	Self-Defined Gender Identity	Age	Years of Transitioning	Medical Treatments
Alex	Trans or Free person	32	7 years	Hormonal treatment
Ana	Woman	15	3 years	Hormonal treatment
Carlos	Man	35	5 years	Hormonal treatment and mastectomy
Carolina	Woman	42	19 years	Hormonal treatment, genital reconstruction surgery and mammoplasty
Daniel	Trans Boy	33	7 years	Hormonal treatment
Iker	Transqueer or transgender	28	6 years	Hormonal treatment
Llurena	Woman	32	7 years	Hormonal treatment and genital reconstruction surgery
Raúl	Man	24	3 years	Hormonal treatment

**Table 2:** Basic themes and organizing themes according to transition stages.

Basic Themes	Organizing Themes	Transition Stages
1.’Male/female’ PAS	Sport preferences	In the closet
2.Nonbinary people preferences		
3. Family support	Playing ‘inappropriate’ sports	
4. Harassment and marginalization		
5. Discomfort and insecurity		
6. Body exposure	Body consciousness	
7. Confrontations		
8. Menstruation		
9. Hyper-vigilance of their bodies	Gaining passing	Opening up
10. Self-control and self-exclusion of certain PAS spaces		
11. Gender technologies		
12. Non-normative PAS spaces		
13. The resistance of visible bodies		
14. Sexual reconstruction surgery	The body as a passport	Reassuring
15. The promise of a better life		
16. Competition or gender transition process. Leaving a dream	Pending barriers	
17. Not enough ‘male’. Biological barriers		

## Data Availability

The datasets analyzed during the current study are available from the corresponding author on reasonable request.
